# Clinical evaluation of haploidentical hematopoietic combined with human umbilical cord-derived mesenchymal stem cells in severe aplastic anemia

**DOI:** 10.1186/s40001-018-0311-3

**Published:** 2018-03-01

**Authors:** Lixin Xu, Zhouyang Liu, Yamei Wu, Xueliang Yang, Yongbin Cao, Xiaohong Li, Bei Yan, Songwei Li, Wanming Da, Xiaoxiong Wu

**Affiliations:** 1 Department of Hematology, The First Affiliated Hospital of Chinese PLA General Hospital, No. 51 Fucheng Road, Haidian District, Beijing, 100048 China; 2 Department of Hematology, Chinese PLA General Hospital, Beijing, 100853 China

**Keywords:** Severe aplastic anemia, Haploidentical hematopoietic stem cells transplantation, Umbilical cord-derived mesenchymal stem cells, Graft versus host disease

## Abstract

**Background:**

This study not only evaluated the clinical effects of treatment using haploidentical hematopoietic stem cells (haplo-HSCs) combined with human umbilical cord mesenchymal stem cells (UC-MSCs) in patients with severe aplastic anemia (SAA), but also investigated the factors related to graft versus host disease (GVHD).

**Methods:**

Cotransplantation of haplo-HSCs and UC-MSCs was performed in 24 SAA patients. The conditioning regimens consisted of rabbit anti-human T-lymphocyte immunoglobulin (ATG), cyclophosphamide, and fludarabine with or without busulfan. GVHD was prevented using cyclosporine A, ATG, anti-CD25 monoclonal antibody, and mycophenolate material.

**Results:**

The incidence of acute GVHD was 50%. The incidence of severe acute GVHD was not related to gender, age, donor-recipient relations, and patient/donor pair, while patient/donor pair (*r* = 0.541, *P* = 0.022) was significantly correlated with incidence of chronic GVHD. Upon follow-up for a median of 13 months, 5 of the 24 patients (20.8%) were dead. The survival rates at 3 and 6 months in all patients were 87.5% (21/24) and 83.3% (20/24), respectively.

**Conclusion:**

Cotransplantation of haplo-HSCs combined with UC-MSCs was an effective and safe approach for the treatment of patients with SAA. The appropriate conditioning regimen and early treatment for infection also played a critical role in the success of HSCT.

**Electronic supplementary material:**

The online version of this article (10.1186/s40001-018-0311-3) contains supplementary material, which is available to authorized users.

## Background

Severe aplastic anemia (SAA) is a life-threatening disease characterized by hypoplastic bone marrow and pancytopenia [1]. Although allogeneic hematopoietic stem cell transplantation (HSCT) is the first choice for the treatment of SAA [2], it is difficult to search for the human leukocyte antigen (HLA)-compatible donors in China [3]. Recent studies have investigated the effect of haploidentical HSCT (haplo-HSCT) in SAA patients, and it has been considered as an optional treatment [4–7]. Nevertheless, the high incidence of graft versus host disease (GVHD) limited the clinical application of haplo-HSCT in patients with SAA.

It has been demonstrated that mesenchymal stem cells (MSCs) can reduce the risks of both acute GVHD (aGVHD) and chronic GVHD (cGVHD) [8]. Notably, human umbilical cord-derived mesenchymal stem cells (UC-MSCs) have higher activities of proliferation and differentiation in comparison with the bone marrow MSCs [9]. Recent study has revealed that UC-MSCs can not only reduce the risk of GVHD, but also increase the transplantation rate of allogeneic HSCs [10]. A previous study by Wu et al. [11] and another research by Zhang et al. [12] have preliminarily shown that the cotransplantation of haplo-HSCs and third-party donor-derived UC-MSCs achieved favorable outcomes for patients with aplastic anemia. However, several problems remain to be further resolved, such as the correlation factors affecting GVHD and lack of optimal sources of donors.

In this study, we reviewed 24 patients with SAA who received the cotransplantation of haplo-HSCs and UC-MSCs with modified conditioning, and evaluated the safety and efficacy of the cotransplantation of UC-MSCs and donor HSCs in SAA patients and investigated the factors related to GVHD.

## Methods

### Patients

The whole protocol was approved by the Institutional Review Board of our hospital, and informed written consents were obtained from all patients. All donors were eligible for donating HSCs, and they also signed the informed consent forms for donation before transplantation.

This study recruited 24 patients with SAA (14 males and 10 females) who underwent cotransplantation of human UC-MSCs and HSCs from June 2010 to August 2013. The inclusion criteria were as follows: patients presenting with SAA or very severe aplastic anemia (VSAA) were defined according to the International Aplastic Anemia Study Group [13]; patients who underwent previous therapy regimens, including cyclosporine A (CSA), stanozole/andriol, granulocyte colony stimulating factor (G-CSF), anti-human T-lymphocyte immunoglobulin (ATG), Erythropoietin (EPO), glucocorticoid—however, they failed to respond to this therapy regimen; patients who received multiple transfusions and whose transfusion dependence was confirmed when the transplantation was performed; patients who agreed to participate in HSCT; and all donors who were relatives to the patients. Patients who had uncontrolled infections and severe liver, renal, lung, or heart diseases before transplantation were excluded in this study. The detailed information about patients and preparation of donors is listed in Additional file 1: Table S1.

### Preparation of UC-MSCs and HSCs

UC-MSCs were purchased from the National Engineering Research Center of Cell Products. HSCs were isolated from peripheral blood of the donors who were injected subcutaneously with recombinant human G-CSF (rhG-CSF, 5 µg/kg day) for 5–6 consecutive days. In brief, peripheral blood (200–600 mL) was collected on day 5, and red cells were removed to avoid the incompatibility of major cross-match. Then, HSCs were separated using Fenwal CS-3000 plus blood cell separator (Baxter International Inc., Deerfield, IL, USA) on days 6 and 7. Finally, mononuclear cells [(4–6) × 10^8^/kg] and CD34^+^ cells [(2–4) × 10^8^/kg] were obtained.

### HSCs and UC-MSCs transplantation

**Fig. 1:**
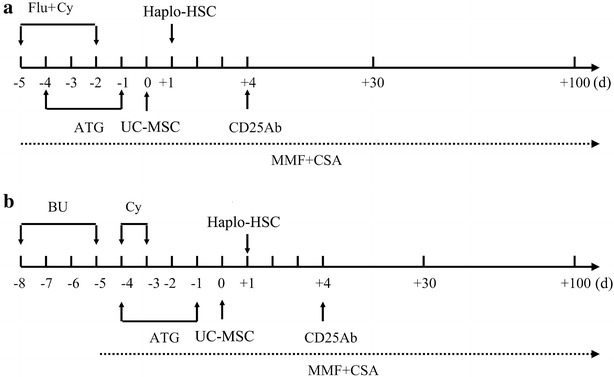
The schematic diagram of conditioning regimen and prophylaxis and management of GVHD. Conditioning included 1 of 2 regimens: for patients with SAA-I (**a**), intravenous administration of fludarabine (Flu) 30 mg/(m^2^ day) and cyclophosphamide (Cy) 500–800 mg/(m^2^ day) from day − 5 to − 2, anti-human T-lymphocyte immunoglobulin (ATG) 5 μg/(kg day) from day − 4 to − 1; or for SAA-II (**b**), the same treatment of ATG and Cy with busulfan (Bu) 0.6 mg/(kg 6 h) from day − 7 to − 6. Prophylaxis and management of GVHD included: intravenous administration of cyclosporine A (CSA) 3 mg/kg from day − 5, followed by gradual decrease in concentration after reaching a target trough blood concentration of 250–450 ng/mL and then withdrawal in the next 2–3 months, oral mycophenolate mofetil (MMF) administration (20 mg/kg/day) from day − 1 to + 100, and intravenous anti-CD25 antibody (CD25Ab) monoclonal antibody (0.5 mg/kg/day) on day + 4 after HSCT. SAA-I: acute severe aplastic anemia, SAA-II: chronic severe aplastic anemia

Anti-inflammatory, hepatoprotective and gastric mucosa-protecting treatments were performed on patients with sodium bicarbonate, dexamethasone and promethazine before transplantation. The conditioning regimens included rabbit ATG (Fresenius AG, Oberursel, Germany), cyclophosphamide (Cy), and fludarabine (Flu) treatment. For the patients with acute SAA (SAA-I), intravenous administration of 30 mg/(m^2^ day) of Flu and 500–800 mg/(m^2^ day) of Cy was performed from days − 5 to − 2, and 5 μg/(kg day) of ATG was administered from days − 4 to − 1 (Fig. 1a). For the patients with chronic SAA (SAA-II), the same treatment of ATG and Cy was applied as above with the supplement of 0.6 mg/(kg 6 h) of busulfan (BU) from days − 8 to − 5 prior to transplantation (Fig. 1b) [3]. On day 0, HSCs were infused intravenously. Then, a total of 5 × 10^5^/kg UC-MSCs was transfused at 4 h before infusion with HSCs.

### Management of GVHD

Prophylaxis of GVHD was carried out by the administration of immunosuppressive agents, such as CSA, mycophenolate mofetil (MMF), and anti-CD25 antibody (CD25Ab; Basiliximab, Novartis Pharma Stein AG, Stein, Switzerland). To achieve a target trough with blood concentration of 250–450 ng/mL at 12 months after HSCT, CSA (3 mg/kg) was conducted by intravenous injection from day − 5 (Fig. 1); then, CSA concentration was gradually decreased and withdrawn in the next 2–3 months. Oral MMF administration (20 mg/kg/day) was conducted from days − 1 to day + 100, and intravenous injection of CD25Ab monoclonal antibody (0.5 mg/kg/day) was performed only on day + 4 after HSCT (Fig. 1). Glucksberg-Seattle criteria (GSC) [14] were used for the diagnosis and grading of GVHD, and those with grade III-IV acute GVHD (aGVHD) were served as severe aGVHD.

### Supportive care

Patients were housed in the laminar flow clean ward. They were asked to take a medicated bath, and treated with acyclovir, ornidazole, sulfamethoxazole trimethoprim, and sulfadiazine 1 or 2 days before conditioning. On day + 3 after HSCT, G-CSF and thrombopoietin (TPO) were administered subcutaneously until the achievement of hematopoietic reconstitution. Prostaglandin E1 (PGE1; 20 μg/day; Beijing Tide Pharmaceutical Co., LTD, Beijing, China) and compound Danshen (20 mg/day; Shanghai No. 1 Biochemical & Pharmaceutical Co., LTD, Shanghai, China) were administered from day − 5 to day + 14 as hepatic veno-occlusive disease (VOD) prophylaxis. Mesna injection (Baxter, Shanghai, China) was used to prevent the hemorrhagic cystitis (HC).

### Engraftment

Neutrophil engraftment was defined as the first of three consecutive days with an absolute neutrophil count (ANC) above 0.5 × 10^9^/L, and platelet engraftment was defined as the first day of a week with the platelet count exceeding 20 × 10^9^/L in the absence of transfusion. Hematopoietic chimerism was assessed using peripheral blood samples of the donor and recipient via short-tandem repeated sequence-PCR (STR-PCR) DNA fingerprinting [15] for sex-matched pairs and karyotype analysis for sex-mismatched pairs. The bone marrow of the recipient was analyzed for hematopoietic chimerism every 30 days until 90 days after HSCT. For blood-type incompatible pairs, the measurement of blood-type titer was processed weekly after hematopoietic reconstitution.

### Statistical analysis

Patients were followed up for 3–44 months. Kaplan–Meier analysis was calculated as survival in the absence of death. The relationships between the incidence of GVHD and gender, age, blood type, donor–recipient relations, and patient/donor pair were evaluated using Chi-square test. All statistical analyses were performed using the standard statistical package of SPSS 19.0 (IBM, Armonk, New York, USA). A two-sided *P* value of 0.05 or less was considered statistically significant.

## Results

### Engraftment and chimerism

Clinical features of patients and their donors, and the subsequent outcomes in 24 case are shown in Table 1. The median scores of infusion numbers of MNCs and CD34^+^ cells were 10.6 × 10^8^/kg and 4.55 × 10^6^/kg for all the patients, respectively. The median times to achieve neutrophil and platelet engraftment were 11 and 13 days, respectively. All the patients achieved 70–100% donor chimerism within 1 month after haplo-HSCT.

**Table 1:** Clinical features of patients and their donors as well as the outcomes of 24 cases

Case	Sex/age	Donor/age	HLA -matched	ABO pairs D/R	MNC 108/kg	CD34^+^ 106/kg	ANC > 2 × 109/L (D)	PLT > 20 × 109/L (D)	aGVHD grade/cGVHD grade	Outcome (M)
1	F/25	Mother/47	3/6	B/AB	9.43	1.9	12	15	–/–	Dead 3
2	M/14	Mother/39	3/6	O/B	10.96	12.83	12	13	–/–	Dead 3
3	M/5	Mother/30	4/6	AB/B	10.2	2.79	12	13	III/Ext	Dead 12
4	M/9	Sister/22	5/6	O/O	10.37	3.24	10	12	IV/Ext	Alive 12
5	M/23	Mother/51	4/6	B/B	8.1	2.97	10	14	–/Ext	Alive 10
6	F/18	Brother/22	3/6	O/O	10.56	4.546	10	14	II/Lim	Alive 13
7	M/16	Father/43	3/6	B/B	8.82	3.25	10	14	III/–	Alive 13
8	M/13	Mother/33	3/6	0/A	10.97	3.47	9	10	III/–	Alive 11
9	M/22	Father/41	4/6	O/A	10.06	3.79	8	14	IV/–	Dead 4
10	F/9	Mother/31	3/6	A/A	9.62	4.71	10	12	–/–	Alive 10
11	M/21	Father/45	3/6	A/A	10.83	4.02	11	13	–/–	Alive 11
12	M/25	Mother/48	3/6	A/AB	8.05	3.45	13	13	II/–	Alive 19
13	F/25	Mother/47	3/6	A/A	12.47	18.65	11	15	III/–	Alive 17
14	F/8	Mother/32	3/6	A/A	10.03	5.33	12	15	I/–	Alive 16
15	M/16	Father/43	3/6	B/B	13.18	7.96	13	15	–/–	Alive 15
16	F/17	Sister/27	3/6	B/B	6.3	3.75	21	25	–/–	Alive 20
17	F/13	Mother/46	4/6	O/AB	9.96	10.2	12	12	I/–	Alive 27
18	M/17	Father/37	3/6	O/O	9.36	7.52	11	13	I/Lim	Alive 41
19	F/24	Sister/21	4/6	A/A	9.66	6.94	11	13	–/–	Alive 41
20	M/20	Sister/23	3/6	B/AB	9.28	5.08	16	18	I/Lim	Alive 44
21	M/15	Mother/37	4/6	O/O	7.93	3.76	8	10	–/–	Dead 3
22	F/13	Sister/18	5/6	B/B	11.2	12.56	10	10	–/–	Alive 4
23	M/8	Mother/32	3/6	A/A	8.1	3.94	10	12	–/–	Alive 5
24	F/55	Brother/49	3/6	O/B	9.24	4.72	10	11	–/–	Alive 12

*F* female, *M* male, *HLA* human leukocyte antigen, *D* donor, *R* recipient, *MNC* mononuclear cells, *ANC* absolute neutrophil count, *PLT* platelets, *aGVHD* acute graft-versus-host disease, *cGVHD* chronic graft-versus-host disease, *Ext* extensive, *Lim* limited, *D* day, *M* months

### GVHD

The GVHD incidences of all patients after HSCT are shown in Table 2. Of the 24 patients, 12 cases (50%) developed aGVHD, including 4 (16.7%) with grade I, 2 (8.35%) with grade II, 4 (16.7%) with grade III, and 2 (8.35%) with grade IV. Meanwhile, 3 of the 24 (12.5%) patients developed extensive cGVHD, and 2 cases among them also experienced III–IV aGVHD. In addition, 3 of the 24 (12.5%) patients who experienced I–II aGVHD also developed limited cGVHD. The other 11 patients had no GVHD. As shown in Table 3, the incidence of severe aGVHD was not related to gender, age, donor–recipient relations and patient/donor pair, while patient/donor pair was significantly correlated with extensive cGVHD (*r* = 0.541, *P* = 0.022). The postoperative incidences of extensive cGVHD were 2/15 of patients with blood-type compatibility, 1/1 of major cross-match mismatch and 0 of minor cross-match mismatch. The rise in GVHD incidence paralleled the increase in HLA loci (*χ*^2^ = 7.764, *P* = 0.022). Extensive cGVHD trended to occur in male and patients who also suffered from severe aGVHD (*P* = 0.060 and *P* = 0.099, respectively).

**Table 2:** GVHD incidences in allogeneic HSCT recipients

Parameters	*N* = 24, (%)
Acute GVHD	
I	4 (16.7%)
II	2 (8.35%)
III	4 (16.7%)
IV	2 (8.35%)
Chronic GVHD	
Lim	3 (12.5%)
Ext	3 (12.5%)

*HSCT* hematopoietic stem cell transplantation, *aGVHD* acute graft-versus-host disease, *cGVHD* chronic graft-versus-host disease, *Ext* extensive, *Lim* limited

**Table 3:** The correlation of variables with GVHD incidence in allogeneic HSCT recipients

Variables	Severe aGVHD			Extensive cGVHD		
	Incidence (%)	*r*	*P* value	Incidence (%)	*r*	*P* value
Gender		0.293	0.134		0.319	0.060
Male	5/14 (35.7%)			3/14 (21.4%)		
Female	1/10 (10%)			0/10		
Age, years		0.05	0.807		0.033	0.872
≥ 20	2/9 (22.2%)			1/9 (11.1%)		
< 20	4/15 (26.7%)			2/15 (13.3%)		
Donor-recipient relationship		0.083	0.599		0.055	0.465
Mother–child	3/12 (25%)			2/12 (16.7%)		
Father–child	2/5 (25%)			0/5		
Siblings	1/7 (14.3%)			1/7 (14.3%)		
Patient/donor pair		0.225	0.566		0.541	0.022
3 HLA loci	3/16 (18.8%)			0/16		
2 HLA loci	2/6 (33.3%)			2/6 (33.3%)		
1 HLA loci	1/2 (50%)			1/2 (50%)		
Severe aGVHD					0.364	0.099
Yes				2/6 (33.3%)		
No				1/18 (5.6%)		

*HSCT* hematopoietic stem cell transplantation, *aGVHD* acute graft-versus-host disease, *cGVHD* chronic graft-versus-host disease, *HLA* human leukocyte antigen

### Other complications

During HSCT period, all patients suffered from nausea, vomiting, and various degrees of anepithymia, and then these symptoms regressed after symptomatic and supportive therapies. Nineteen patients (19/24, 79.2%) who developed stomatitis also showed improvement after mouth care and the topical application of epithelium growth factor. At the stage of bone marrow suppression, various degrees of fevers occurred in patients, and then fevers were controlled by the administrations of imipenem, vancomycin, and antifungals. The incidence of pulmonary infection was 16.7% (4/24). Two cases with pulmonary infection developed into severe pneumonia, and one of them finally died. There were two patients with septicemia and two cases with engraftment syndrome. Fifteen patients (15/24, 62.5%) had viral infections, including Cytomegalovirus (CMV) or Epstein Barr Virus (EBV)-emia in eight cases, and two or more viruses infection in five cases. Three patients (3/24, 12.5%) developed HC. Sixteen patients suffered from diarrhea, and they all responded well to antidiarrheal treatment and regulation of intestinal microecology. The incidences of infectious diarrhea, fungal enteritis, and viral enteritis were 29.2% (7/24), 4.2% (1/24), 16.7% (6/24), respectively, and all of these cases developed into grade III-IV intestinal GVHD. Besides, three patients developed epilepsy. Among them, seizure was resolved in one patient since day + 28 via the replacement of CSA by FK506, and this patient was in stable condition in 2 years after being discharged. The second patient with refractory seizures since day + 60 was diagnosed as positive EBV in his cerebrospinal fluid, and then the patient’s condition improved after antiviral therapy. However, drop of blood cells and infection occurred in the third patient with seizures on day + 90 of haplo-HSCT, and he refused further treatment and finally died.

### Follow-up

**Fig. 2:**
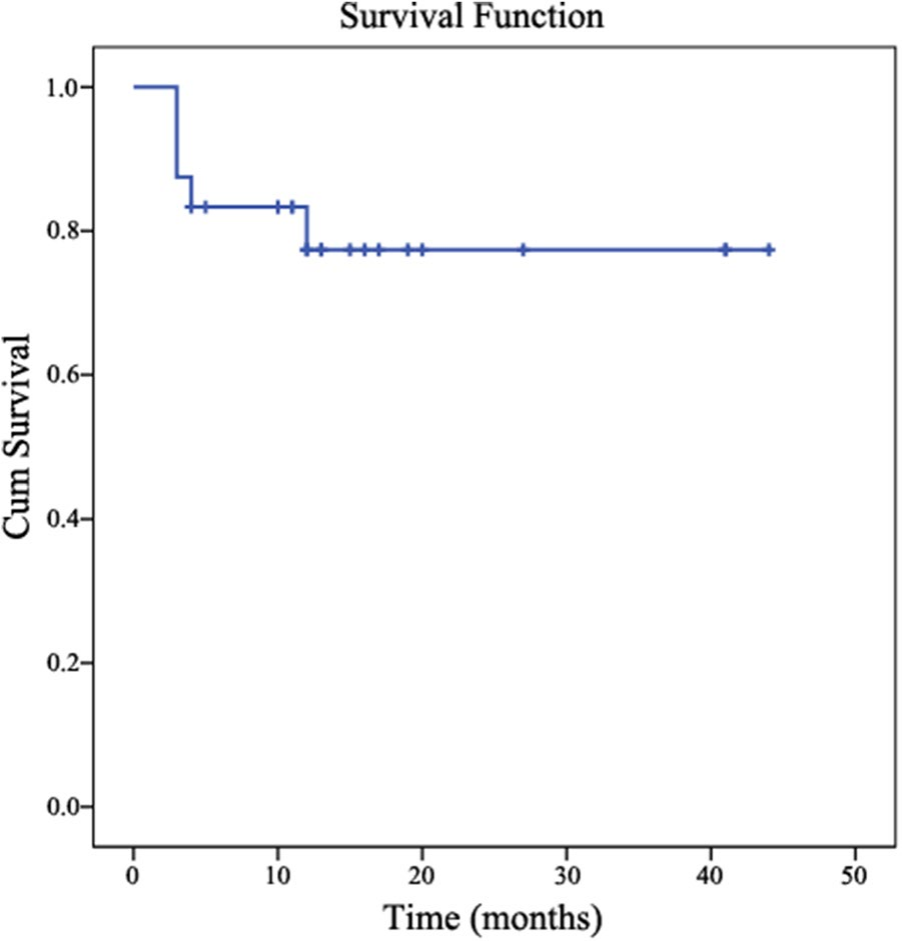
Survival curves of patients with SAA after cotransplantation of haploidentical HSCs and human UC-MSCs. This curve was generated using Kaplan–Meier analysis. The survival rate at 44 months in all the patients was 79.2%

Upon follow-up for a median of 13 months, 5 of the 24 patients (20.8%) were dead. The survival rates at 3 and 6 months in all patients were 87.5% (21/24) and 83.3% (20/24), respectively (Fig. 2). Among the five dead patients, two died as a result of early graft rejection; one died because of rejecting further treatment for reduced blood cell counts and pulmonary infection; the other two patients died due to the uncontrollable grade IV aGVHD, among whom one also suffered from serious pulmonary infection 1 year after haplo-HSCT.

## Discussion

Recent studies suggested that immunosuppressants improved the efficacy of HSCT in patients with SAA [16, 17]. In HLA-matched transplantation for treatment of aplastic anemia, the conditioning regimen consisting of Cy, ATG, and Flu could remarkably decrease the failure rate of transplantation [18, 19]. Similarly, our study also selected this conditioning regimen in patients with SAA-I; however, we adjusted Cy at the concentration of 500–800 mg/m^2^ day according to the demographic data of the patients. For patients with SAA-II, reduced doses of BU was supplemented into the conditioning regimen to enhance immune ablation. Our results demonstrated that all patients with modified conditioning regimen achieved a high engraftment without serious toxic reactions or deaths.

GVHD, as a common complication following allogeneic HSCT, was the principal cause of morbidity and nonrelapse mortality in long-term survivors [20, 21]. Our study showed that the incidence of aGVHD after haplo-HSCT was 50% (six cases of grade I–II aGVHD and six cases of grade III–IV aGVHD), which was consistent with Wu’s study showing 57.1% incidence of aGVHD [3]. However, previous studies showed the incidence ranged of 44–64% for II–IV aGVHD and 12–26% for extensive cGVHD [22, 23]. Therefore, it seemed that the infusion of third-party donor-derived UC-MSCs might play a considerable role in reducing the incidence of severe GVHD. Furthermore, our study found that gender, age, donor-recipient relationship, and patient/donor pair were not associated with the incidence of the severe aGVHD, while the incidences of extensive cGVHD were increased in patients with major cross-match mismatch and less HLA loci. Besides, cGVHD incidences were also more likely to occur in males or patients who had developed aGVHD. All these results suggested that it might be an optional prevention route for cGVHD to effectively control severe aGVHD and avoid major cross-match mismatch.

This study showed that the mortality for the entire group of patients was 20.8%, suggesting that cotransplantation of haplo-HSCs and human UC-MSCs might be an effective treatment modality for patients with SAA. However, despite infusions of human UC-MSC after allogeneic HSCT, Si et al. [24] reported that the 3-year overall survival rate was 74.2%, which was lower than our results (87.5%). The differences in the survival rate might be attributable to the pediatric patients enrolled in Si et al.’s study. Besides, the deaths reported in five patients in this study all occurred due to different complications, such as early graft rejection, reduced blood cell counts, pulmonary infection, and aGVHD, which indicated that preoperative prophylaxis and care also played essential role in a successful HSCT.

This study has some limitations. First, because cotransplantation of human UC-MSCs and HSCs is not a conventional treatment for patients with SAA, it was difficult to recruit the patients who underwent cotransplantation of human UC-MSCs and HSCs for this study. Due to the small sample size of this study, a prospective study with larger sample size will be performed to confirm our results. Second, a thorough evaluation of the patients to determine the correlation factors affecting GVHD was still required to conduct the cotransplantation. Third, no control group to compare the efficacy and safety of cotransplantation of UC-MSCs and haplo-HSCs was available. Thus, a case–control group comparison of evaluations of treatments with HSCs or UC-MSCs alone is necessary to strengthen the integrity and statistical power of this study and to validate our present results in the future study. Despite these limitations, this study has preliminarily demonstrated the safety and effectiveness of cotransplantation of haplo-HSCs and UC-MSCs for the treatment of patients with SAA.

## Conclusion

This study suggested that the combined application of UC-MSCs in HSCT was a safe and efficacious method for the treatment of SAA. The appropriate conditioning regimen and early treatment for infections and other complications also played a critical role in the success of HSCT.

## Additional file

**Additional file 1: Table S1.** Pre-transplant characteristics of patients and their donors.

## Abbreviations

UC-MSCs: umbilical cord mesenchymal stem cells
SAA: severe aplastic anemia
GVHD: graft versus host disease
HSCT: hematopoietic stem cell transplantation
HLA: the human leukocyte antigen
aGVHD: acute GVHD
cGVHD: chronic GVHD
VSAA: very severe aplastic anemia
CSA: cyclosporine A
G-CSF: granulocyte colony stimulating factor
ATG: anti-human T-lymphocyte immunoglobulin
EPO: erythropoietin
Cy: cyclophosphamide
Flu: fludarabine
MMF: mycophenolate mofetil
TPO: thrombopoietin
PGE1: prostaglandin E1
VOD: veno-occlusive disease
ANC: absolute neutrophil count
STR-PCR: short-tandem repeated sequence-PCR
CMV: cytomegalovirus
EBV: Epstein Barr Virus
