# Supplementary material for: Clinical evaluation of haploidentical hematopoietic combined with human umbilical cord-derived mesenchymal stem cells in severe aplastic anemia
Source: Eur J Med Res. 2018 Mar 1;23:12. doi: 10.1186/s40001-018-0311-3 (PMC5831581; doi:10.1186/s40001-018-0311-3)
Supplement: Supplementary file 1 — Additional file 1: Table S1. Pre-transplant characteristics of patients and their donors. [file 40001_2018_311_MOESM1_ESM.docx]

**Supplemental Table 1** Pre-transplant characteristics of patients and their donors.

| Parameters | N = 24 |
| --- | --- |
| Patient age (years) |  |
| Median | 13 |
| Range | 5-55 |
| Gender |  |
| Male | 14 |
| Female | 10 |
| Status prior to co-transplantation |  |
| SAA-I | 16 |
| SAA-II | 8 |
| Donor age (years) |  |
| Median | 37 |
| Range | 12-49 |
| Patient/Donor pair |  |
| 3 HLA loci | 16 |
| 2 HLA loci | 6 |
| 1 HLA loci | 2 |
| ABO pairs |  |
| Compatibility | 15 |
| Major side blood group mismatch | 1 |
| Secondary side blood group mismatch | 8 |
| Donor-recipient relationship |  |
| Mother-child | 12 |
| Father-child | 5 |
| Siblings | 7 |
| Sex pairs of the donor-recipient |  |
| Male-female | 9 |
| Male-male | 5 |
| Female-female | 8 |

SAA-I: acute severe aplastic anemia; SAA-II: chronic severe aplastic anemia; HLA: human leukocyte antigen.
